# Foreign

**Published:** 1841-01-23

**Authors:** 


### FOREIGN.

*M. Piorry on Hæmitis.*—The term *hemite* has been given to that alteration of the blood which exists in inflammatory diseases, and which is characterized by the formation of the buffy coat on its surface when it has been drawn and allowed to rest. This condition of the blood has been recognised from the earliest period; but it has only been within the last few years that the constitutional disturbance occasioned by it, before any decided local inflammation has been set up, has been attended to as a special disease. The "inflammatory fever," and "Synocha," of older authors, the "Angeiotenic fever" of *Pinel*, and the "Rheumatic fever" of *Sarcone*, exhibit, it must be admitted, most of the symptoms which exist in genuine "hæmitis;" but the latter term is greatly preferable as being much more explicit, and as expressive of the pathological cause of the disease.

It is but fair to acknowledge that *Sydenham*, *Van Swieten*, *Boerhave*, and many other excellent physicians of the old school, seem to have been much better acquainted with this malady than the authors of more recent times: it is well known how constantly allusion is made in their writings to the overplasticity of the blood causing obstructions in the smaller vessels, and thus giving rise to inflammation and its consequences—a doctrine which *Magendie* and some of the German writers of the present day have been endeavouring to re-establish.

After alluding to the influence of the size, depth, &c., of the vessel, into which the blood is received, on the production of the buffy coat—which, by the bye, has been much exaggerated by many—M. Piorry goes on to state:—"In 1826 I published an account of numerous experiments to prove that *the serum of the blood contains the materials of the buffy coat, and that this is formed by precipitation.* More recently it has been found by microscopical examination, that it (the buffy coat) is composed of colourless globules of an albuminous or fibrinous nature."

It has been objected to M. Piorry's views on hæmitis, that the blood is not "an organ," and cannot therefore be susceptible of inflammation; to this he replies, "Bordeu has triumphantly answered this objection. In treating of the medical analysis of the blood, which he calls fluid flesh (chair coulante) he views it as so completely a living organ that he rejects all the analyses of mere chemists, and asserts that it is a physician only that can examine the blood properly. And is it not the case that, in every inflamed part, we must take into account alike the fluids and the solids; in other words, alike the blood-vessels and the blood itself?"

M. Piorry proceeds to describe the appearances exhibited by the blood, in cases of hæmitis, during coagulation:—

"The two elements of the blood separate, the coagulum falling to the bottom, and the serum resting uppermost; in other cases this is reversed: the separation takes place usually eight or ten minutes after the blood has been drawn from the veins. The serosity is not at first clear and transparent, but it is of a grayish opaline or pearly white colour; sometimes it is yellowish. The surface of the coagulum is at first uniformly red, but this soon begins to exhibit a grayish stratum, which is gradually deposited more and more as the serum becomes clearer, and loses its opaque opaline appearance. The buffy coat is thus formed because the serum is uppermost, and floats above the serum." . . . . . . . "The buffy coat adheres to the coagulum; its thickness varies; its colour is yellowish, grayish, and sometimes has a slightly green hue; occasionally it is mixed with some blood globules. Its consistence varies exceedingly in different cases; it is always the greatest at the surface: the supernatant serum is generally clear, transparent, and of a citrine colour." . . . . . . . "If we remove with a syphon the serum from inflamed blood, as it separates from the coagulum, and put it on a serous membrane, or indeed upon a glass, a deposit similar to the *couenne*, or buffy coat, will be observed to take place. This deposit is sometimes in a mass; at other times it separates into two portions, of which one falls to the bottom, and the other mounts to the surface. If it be exposed to the air, it reddens (?) and resembles a good deal the fibrine precipitated from the blood when this is briskly stirred about with a cane upon being drawn from a vein." . . . . . . . "The examination of the buffy coat with the microscope and with chemical means has shown that the plastic lymph is formed from the serosity of the blood, and is composed of globules."

*Necroscopic appearances.*—"Most of the physical appearances exhibited by the blood after death are the same as what we have mentioned are observed in it when drawn from the veins during life; this fluid is more or less extensively coagulated in the heart and blood-vessels. Laennec, Legroux, and Bouillaud, have described with much care the appearance of the coagula often found in the cavities of the heart and of the larger veins; occasionally, too, in one of the larger arteries. Another necroscopic sign of hæmitis is the occurrence of a layer of plastic lymph, very similar to the buffy coat of the blood, on the surface of some of the serous membranes, as the pleura, pericardium, peritoneum, and the synovial capsules. Sometimes also a blistered surface is found covered with a layer of dense coagulable lymph: and occasionally also we observe in the cellular tissue around any inflamed part a dense resisting layer of this lymph, which has been mistaken for a scirrhous formation.

Besides these signs, the lining membrane of the heart and blood-vessels often exhibits a most distinct vascular congestion."

*Cause.*—"In nine-tenths of the cases of hæmitis, the disease may be traced to the influence of cold, especially where the surface has been perspiring. Every one knows how almost invariably pneumonitis, and hæmoarthritis, are attributable to this cause. Now let us remember that an inflamed state of the blood in a vast majority of cases precedes the invasion of the local symptoms, and is indicated by a feeling of general *malaise*, tendency to shivering, weariness, and pains in the limbs and back, &c.

M. Piorry doubts that excesses in eating are apt, as supposed by many people, to induce hæmitis; they have a tendency, says he, to cause rather polyhyperhæmia than hæmitis. Even the inordinate use of wine and spirituous drinks seldom seems to bring on this condition of the blood, unless the agency of cold be exerted on the body at the same time.

Disturbance of the respiratory function appears often to have a tendency to bring on an inflamed state of the circulating fluid; hence, perhaps, the frequency of fibrinous concretions in the heart in cases of anhæmatosis.

*Symptomatology.*—It is difficult to describe the symptoms of hæmitis, apart from those of its complications—in other words, of the local inflammation which is almost invariably very quickly induced by it. One of its earliest symptoms is a shivering, which is generally more or less severe and lengthened in proportion as the disease is more or less decided; this is succeeded by a heat over the whole surface; the pulse becomes much quickened, the face is flushed, and the capillary vessels in every part seem to be highly injected. But it is unnecessary to pursue this part of the subject, as the description given by M. Piorry corresponds exactly with the symptoms of synocha or inflammatory fever of other authors. That he really regards these diseases as perfectly analogous is evident from what he says as to the rapid course of the slighter cases of *hæmitis:*

"If the hæmitis be slight, and if it will terminate favourably after a few days' rest, a gentle diarrhœa comes on, or the patient discharges a quantity of sedimentary urine, and the health is gradually restored:" and again, "at the commencement of all exanthematous fevers, during the whole period which precedes the appearance of the eruption, the symptoms of hæmitis are present, and yet there is no buffy state of the blood present."

Now for a few words as to the treatment recommended by M. Piorry in hæmitis. Blood-letting, as a matter of course, he enjoins. Purgatives he does not approve of; "they not only withdraw the serosity of the blood, but they irritate the intestine; a few glasses of Seidlitz water, or an enema occasionally, are best. (How practical!) Blisters are decidedly useful, but not at the commencement of hæmitis; it is towards the close of the attack, and especially when there is hydrohæmia or anæmia, that they are most beneficial. One of the most efficacious remedies in hæmitis is the copious use of diluents: "we can best diminish the plasticity of the blood, by making the patient drink very freely of any mild fluid; the blood becomes thus more and more diluted, and less disposed to coagulate."* To facilitate the absorption of the fluid into the blood, our author recommends at the same time the repeated administration of emollient enemata.(!) M. Piorry has no faith in any of the antiphlogistic remedies which have been supposed to counteract the over-plasticity of the blood:—"We have tried to prevent the formation of the *couenne* by the administration of the alkaline carbonates, the preparations of antimony, and various other chemical salts; none of these remedies has been of much advantage; we must receive with extreme reserve many of the statements of Rasori and his followers as to the efficacy of antimonials in the treatment of phlogistic diseases."—*Medico-Chirurgical Review, from Gazette des Medecins-Praticiens.*

*Remarks.*—Much of what M. Piorry has written on hæmitis, or the inflammatory condition of the blood, is strictly true; although unfortunately he has, like most authors on their peculiar or favourite subject, carried his ideas much beyond what is warranted by experience. That the blood is apt under certain circumstances, and more especially in certain habits of body, to acquire an abnormal degree of plasticity, or in other words to contain an unusual quantity of fibrinous or coagulable matter, cannot be disputed by any one: it is a fact of daily occurrence; and it is equally certain that this over-plastic state of the blood is a very general *precursor* of all decidedly inflammatory diseases. It is a common mistake to suppose that the invasion of these diseases is often quite sudden, and is not preceded by any appreciable symptoms. We believe that in almost every case of decided phlegmasia the blood has been for several days, if not weeks, previous to the explosion (so to speak) of the local mischief, in a more or less altered state—a state corresponding with what M. Piorry has designated hæmitis. Many a severe attack might be, no doubt, prevented, if this precursory state was detected sufficiently early, and means were used to impoverish and attenuate the state of the circulating fluids. We cannot indeed suppose that the blood can become so quickly charged with an excess of fibrine, as the suddenness of many an inflammatory attack may lead us to imagine: the train had been laid for some time before the explosion took place.

This inflammatory state of the blood is indicated by the following symptoms: general oppression and weariness after exertion and eating; occasional headach and dulness of the intellect; slight confusion of sight, and, perhaps, tinnitus aurium; occasional nausea and sickness; scanty secretion of the urine, which is almost always deep coloured and hot; flying pains in the joints and limbs; heavy and disturbed sleep, &c. Now, this state of things may continue for weeks, and even months, before any local or well defined attack appear, and all the time the person may have been able to be going about and attending to his affairs,

* M. Piorry remarks that if "we were to be guided by the results of mixing water with the serum of inflammatory blood out of the body, the employment of large quantities of diluents in hæmitis might be deemed any thing but prudent; for if we add pure water to the serum, there is a sudden coagulation induced." "But we must remember," he adds, "that diluents when swallowed reach the blood only after having been sufficiently elaborated."

as usual. Suddenly, as is imagined, he is seized, generally after exposure to damp and cold with shivering, followed by heat, and then the symptoms either of pneumonia, or acute rheumatism, pericarditis, &c., set in.

But if the truth were known, the attack has been by no means sudden; many a warning had been given to the patient that there was something not altogether right with him, and he in all probability had been trying to shake off his unpleasant symptoms, not by lowering his diet, but rather by taking an additional quantity of stimulus in the form of wine, porter, &c. In this manner the *hæmitic* state of the circulating fluid gradually establishes itself, undiscovered alike by the patient, and probably also by his medical attendant. But we cannot at present extend these remarks. Suffice it therefore to say, that although we cannot agree with M. Piorry that every case of synocha is dependent upon hæmitis, we very willingly admit the practical value to be derived from a greater attention to the state of the blood as the groundwork or precursory stage of many inflammatory diseases; more especially of acute rheumatism, pneumonia, and pericarditis.

The first of these diseases—acute general rheumatism or rheumatic fever, as it is often called—is perhaps of all the one which is most essentially connected with that state of blood in which there is a great excess of the fibrinous portion; and it is more than probable that it is from this circumstance that we are to account for the very intimate dependence of pericarditis and many lesions of the heart and great blood-vessels on previous attacks of acute rheumatism. We thus more and more distinctly perceive the high importance of paying extreme attention to the cardiac symptoms during and after (not for weeks only but for months and years) an attack of severe rheumatism.

One word before we close, as to the treatment of *hæmitis*; for it is in this branch of his theme that the observations of M. Piorry, as indeed is the case with most of the medical writings of his countrymen, are most faulty. For example, he does not even once allude to the undisputed effects of mercury upon the over-fibrinous state of the blood; and yet we believe that most English practitioners would rather dispense with the lancet itself than with this most potent remedy in the treatment of hæmitis. Iodine and its preparations exert a similar effect. How far colchicum has any direct influence on the condition of the blood, we cannot say; but it seems probable that it has.

Once more, we strongly urge upon our readers' attention the importance of keeping an eye for a length of time upon such of their patients as have suffered from those inflammatory diseases in which the blood has been highly charged with fibrine; as the foundation of many organic diseases, especially in the thoracic viscera, is often imperceptibly laid during the following six or twelve months.—*Rev.*

The inflammatory condition of the blood is attracting much notice. In the last volume of the Examiner, numerous cases are given in the clinical lectures of Dr. Gerhard, illustrative of cases of this description, which coincide with rheumatism and pneumonia, or rather simple endocarditis. In fact, the existence of endocarditis presupposes, in nearly every case, an inflammatory condition of the blood. The remarks of the reviewer, that, next to blood-letting, mercury is the most directly antiplastic remedy known, are borne out by practice; in fact, blood-letting alone, although sufficient for the cure of most cases, is much less efficient if prescribed singly, than when combined with the mercurials, and often fails entirely.—*Eds.*

---

*On the New (decimal) System of Weights in France.*—Since the beginning of the present year, the French government has ordered that the old system of weights used by chemists and druggists, and consisting of grains, scruples, drachms and ounces be abolished, and be replaced with a decimal system as follows.

| | | | |
|---|---|---|---|
| 1 grain | equiv. to | 5 | centigrammes. |
| 10 grains | ,, | 50 | ,, |
| 20 ,, | ,, | 1 | gramme. |
| 30 ,, | ,, | 1 | gram. & 50 centigrammes. |
| 40 ,, | ,, | 2 | grammes. |
| 50 ,, | ,, | 2 | grams. & 20 centigrammes. |
| 1 scruple (24 gr.) | | 1 | gram. & 30 centigrammes. |
| 1 drachm | ,, | 4 | grammes. |
| 2 drachms | ,, | 8 | ,, |
| Half ounce | ,, | 15 | ,, |
| One ounce | ,, | 30 | ,, |

It will be observed that there is an exact analogy between this decimal system of weights and the existing division of money in France. Thus the *franc*, consists of 20 sous or of 100 centimes, just as the *gramme* consists of twenty grains or of 100 centigrammes.

By bearing this simple rule in mind, the medical practitioner and druggist will at once be able to reduce the former weights to the present decimal standard. Thus, if he wishes to prescribe 12, 15 or 18 grains of a medicine, he has only to substitute the idea of sous for grains; and by reducing the sous into centimes he may reduce, by this simple operation, the grains of any medicine which he may wish into centigrammes.

Thus twelve grains make 60 centigrammes, 15 grains 75 centigrammes, 18 grains 90 centigrammes, 24 grains 1 gramme and 20 centigrammes; just as in 12 sous there are 60 centimes, in 15 sous 75 centimes, in 18 sous 90 centimes, and in 24 sous 1 franc and 20 centimes. Moreover, we have only to substitute the idea of grammes for that of francs, to discover at once the number of grammes which any number of grains compose. Thus 100

grains make five grammes, as 100 sous make five francs; 530 grains make 17 grammes, and 50 centigrammes, as 530 sous make 17 francs and 50 centimes.

The decical numeration is therefore by no means difficult to keep in mind, and by rejecting all other denominations but the two of *grammes* and *centigrammes* the memory is not at all perplexed. In place of using, as hitherto, the term *decagramme* to denote ten grammes, and then speaking of one, two, three decagrammes, it is far better to say at once 10, 20, and 30 grammes; and in the same way, instead of using the term *decigramme*, as has often been done, to denote 10 centigrammes, we have only to say 10 centigrammes at once, and so on, as 20, 30, 40, 50 centigrammes in place of one, two, three, four, and five decigrammes.

By this simple mode of numeration, we reduce the scale of weights to tally with the decimal scale of measures which has been established for a length of time, and is now perfectly understood by every one. The *metre* is, it is well known, the primary or standard measure of length; but its multiple *decametre* and its fraction *decimetre*—denoting the one 10 metres, and the other the 10th part of a metre—are now no longer used. For example, we do not talk of a man's height being one metre and seven decimetres, but one metre and seventy centimetres, nor of any thing costing one sous and 7 decimes, but one sous and 70 centimes.

A few words on the scruple, drachm, (*gros*,) and ounce.

A *scruple* represents 24 grains: to reduce this to the present decimal standard we have only to remember the value of 24 sous; and as they make one franc and 20 centimes, so the scruple is equivalent to one gramme and 20 centigrammes.

The value of the drachm (72 grains) calculated on the bases of five centigrammes to one grain, would be three grammes and 60 centigrammes; but as its value is, according to the *poids de marc*, 3 grammes and 82 centigrammes, and, according to the Pharmacopœia, three grammes and 90 centigrammes, it has been determined to fix it now as an equivalent of an entire number, viz. four grammes or 80 grains. Nothing can therefore be more easy than to reduce drachms into grammes: we have only to multiply the number of the former by four: thus two drachms of any medicine are equivalent to eight grammes, three drachms to twelve grammes, and so forth.

The value of the ounce has been differently estimated. According to the *poids de marc*, it is equal to 30 grammes and 59 centigrammes; according to the Pharmacopœia to 32 grammes; according to the metrical pound to 31 grammes and 25 centigrammes. At the suggestion of M. Double, in his luminous report submitted to the Academy of Medicine, it has now been fixed at the rate or value of 30 grammes exactly, excluding thus the additional 59 centigrammes, or about 11 grains of the *poids de marc* standard. To reduce the ounce into grammes is very simple: it need only be multiplied by 30, or if you prefer by 3, adding a cypher to the product. Thus four ounces of any medicine are equivalent to 120 grammes, six ounces to 180 grammes, and so forth.

Hitherto all has been abundantly simple; but a difficulty remains. This is to fix the value of the pound. If the ounce be 30 grammes, and 16 ounces be still considered as equivalent to one pound, the latter must be rated at 480 grammes; but, for various considerations which we cannot afford space at present to canvass, it has been judged better to rate it at 500 grammes by which valuation it is rendered equivalent to 16 ounces and 20 grammes.—*Ibid, from Bulletin Generale de Theraqeutique.*

The frequent reference to these weights, in in French works, render a knowledge of them important to our readers.

---

### THERAPEUTICS.

*Mr. Donovan on Cod-Oil.**—It appears that cod-oil has been a good deal used, of late, in France and Germany, in certain scrofulous cases. They *say* that when properly administered cod-oil cures scrofula of the bones, marasmus, and chronic arthritis of a scrofulous or rheumatic form. Caries, accompanied by a sore and swelling of the soft parts, requires the treatment with oil to be seconded by local applications, such as compression, and iodurette alcoholic fomentations, cod-oil is of no avail against gouty arthritis, or swelling of any lymphatic glands but those of the abdominal cavity; its action seems doubtful or null in scrofulous phthisis when at all advanced. To produce advantageous results, in any disease, the use of cod-oil must be persevered in for several months, in doses of three or four tablespoonfuls daily.

Now, if this be all true, cod-oil is no bad thing, and it would be well to have it as good as can be got. Perhaps it should *not* taste exactly like train-oil, as that might make one sick, if it did nothing else. So Mr. Donovan has perfected the process for its preparation, and made cod-oil a very respectable oil to take.

Take, says he, any quantity of livers of cod, throw them into a very clean iron pot, and place it on a slow fire, stir them continually until they break down into a kind of pulp: water and oil will have separated. When a thermometer plunged in the pulp will have risen to 192°, the pot should be taken from the fire, its contents transferred to a canvass bag, and a vessel placed underneath. Oil and some water will run through. After twenty-four hours, separate the former by decantation, and filter it through paper.

This oil, thus prepared, is of a pale yellow

*Dublin Journal, July 1, 1840.

colour; its smell is weak, and resembles that of a cod boiled for the table when in excellent condition. Its taste is bland, by no means disagreeable, and as might be expected, is totally free from rancidity. It is very liquid. Its specific gravity in my trials was 0.934, although in all the published tables of specific gravities it is stated to be 0.923. In cold weather it deposits much stearine, and this ought not to be separated.

The product of pure oil is very variable. He has obtained so much as a gallon (wine measure) from twenty-eight pounds of livers, the produce of fifty cods. Sometimes the livers will afford much less. The runnings of the first heat only should be used: a second heat will supply more oil, but it will be comparatively strong-smelling, ill-tasted, and deep-coloured. The above estimate is true only when the fish is in the best season, and fully grown. Towards the close of the season the produce will be less. The livers of some cods are flaccid and lie flat without plumpness on a plane surface. These afford a deficient quantity of oil, a brown, strong-smelling quality, and a large portion of brown water: they are totally unfit for use, and their oil is disgusting. The livers are often found diseased and dark-coloured; such afford a very bad oil, and are of course to be rejected.

On the west coast of Ireland, they consider the beginning of the year the best season, and on the east the month of November for the cod-livers. Thus, concludes Mr. Donovan, in preparing cod-oil fit for medical purposes, three chief things are to be attended to: the livers must be perfectly healthy; they must be as fresh as possible, the least putrescency being injurious; and the heat at which the extrusion of the oil is effected must not exceed 192°.

Cod-liver oil is rarely used in this country. Indeed, the disgust caused by it is a strong reason against its employment. The evidence, however, of its beneficial effects, given by the medical men of Germany, is sufficient to warrant a fair trial.

*Sulphate of Quinine in Enlargement of the Spleen, and in Dropsies after Agues.*—Dr. Levy, physician of the Military Hospital of Val-de-Grace at Paris, has very satisfactorily shown that the dropsical effusions, which not unfrequently supervene upon long neglected agues, are most successfully treated with quinine. He alludes to the researches of MM. Bally, Nonat, Piorry, and other contemporaneous physicians, which have clearly established the superior efficacy of this remedy—to be associated in most cases with the application of the cupping instruments over the left hypochondrium—in dispersing the enlargements of the spleen which so very generally, nay almost always, accompany old intermittent fevers. Indeed, he regards this, the administration of quinine in such cases, as one of the most valuable therapeutic discoveries of recent times. Now, as the dropsical effusions are almost invariably connected with an *infarcted*—to use an old word—state of the spleen, it is a rational deduction to anticipate that the remedy, which is so decidedly efficacious against the *cause*, may not be without its influence upon the *effect*. Certain it is that the use of the ordinarily-resorted-to means, such as diuretics and purgatives, seldom succeeds in dissipating the dropsies which we are at present considering, whereas they may be often dispersed by quinine in full doses.—*Medico-Chirurgical Review, from Gazette Medicale.*

It is rather odd that this should come to us in the guise of a new discovery. Intermittent fever is rather a rare disease in the north of France, and it is not surprising that many points connected with it should from time to time present themselves wtth an air of apparent novelty; to us, however, the subject is a familiar one. The engorgements of the spleen are removeable by quinine, not from any specific action of the remedy on the local affection, but because it cures the general disease on which the enlargement depends. Hence, in most cases, topical remedies, such as cups, if the local disorder is at all inflammatory, or blisters, if chronic, add very much to the good effects of quinine.

In fact, intermittent fever is not cured when the paroxysm is for a time arrested; as long as the diseased condition of the blood continues, the mischief still persists. The best test of the continuance of the disease is the colour of the skin, as indicative of an anemic state of the economy; this is not infallible, but is a tolerably sure guide. As an adjunct to the quinine, we often prescribe the freshly prepared carbonate of iron in doses of ten to twenty grains in the day. Mercurials are, on the whole, productive of much more mischief than good in this state of things, although, in the diseases of the liver dependant upon intermittent, they are often the best remedy. But, even in the latter case, they may be abused, and increase the chronic engorgement of the liver, which they are designed to correct. There is no certain test of their action, judging a priori, except that they are not suited to those cases in which the blood of the patient is deficient in red globules; they act more certainly when there is more positive evidence of inflammatory action.

